# Emerging topics in nanophononics and elastic, acoustic, and mechanical metamaterials: an overview

**DOI:** 10.1515/nanoph-2022-0671

**Published:** 2023-01-27

**Authors:** Anastasiia O. Krushynska, Daniel Torrent, Alejandro M. Aragón, Raffaele Ardito, Osama R. Bilal, Bernard Bonello, Federico Bosia, Yi Chen, Johan Christensen, Andrea Colombi, Steven A. Cummer, Bahram Djafari-Rouhani, Fernando Fraternali, Pavel I. Galich, Pedro David Garcia, Jean-Philippe Groby, Sebastien Guenneau, Michael R. Haberman, Mahmoud I. Hussein, Shahram Janbaz, Noé Jiménez, Abdelkrim Khelif, Vincent Laude, Mohammad J. Mirzaali, Pawel Packo, Antonio Palermo, Yan Pennec, Rubén Picó, María Rosendo López, Stephan Rudykh, Marc Serra-Garcia, Clivia M. Sotomayor Torres, Timothy A. Starkey, Vincent Tournat, Oliver B. Wright

**Affiliations:** Engineering and Technology Institute Groningen, University of Groningen, Groningen 9747AG, The Netherlands; GROC-UJI, Institut de Noves Tecnologies de la Imatge, Universitat Jaume I, Castelló de la Plana 12071, Spain; Faculty of Mechanical, Maritime and Materials Engineering, Delft University of Technology, Delft 2628 CD, The Netherlands; Department of Civil and Environmental Engineering, Politecnico di Milano, Milan 20133, Italy; Department of Mechanical Engineering, University of Connecticut, Storrs, CT 06269, USA; Institut des Nanosciences de Paris, Sorbonne Université, UMR CNRS 7588, Paris 75005, France; DISAT, Politecnico di Torino, Torino 10129, Italy; Institute of Nanotechnology, Karlsruhe Institute of Technology (KIT), 76128 Karlsruhe, Germany; IMDEA Materials Institute, Getafe, Madrid 28906, Spain; Department of Civil, Environmental and Geomatic Engineering, ETH Zürich, Zürich 8093, Switzerland; Department of Electrical and Computer Engineering, Duke University, Durham NC 27708, USA; Institut d’Electronique, de Microléctronique et de Nanotechnologie, UMR CNRS 8520, Université de Lille, Villeneuve d’Ascq 59655, France; Department of Civil Engineering, University of Salerno, Fisciano 84084, Italy; Faculty of Aerospace Engineering, Technion – Israel Institute of Technology, Haifa 32000, Israel; Catalan Institute of Nanoscience and Nanotechnology (ICN2) CSIC and BIST, Barcelona 08193, Spain; Laboratoire d’Acoustique de l’Université du Mans (LAUM), UMR 6613, Institut d’Acoustique – Graduate School (IA-GS), CNRS, Le Mans Université, Le Mans 72085 Cedex 09, France; UMI 2004 Abraham de Moivre-CNRS, Imperial College London, London SW7 2AZ, UK; Walker Department of Mechanical Engineering, The University of Texas at Austin, Austin TX 78712, USA; Ann and H.J. Smead Department of Aerospace Engineering Sciences, University of Colorado Boulder, Boulder CO 80303, USA; Machine Materials Lab, Institute of Physics, University of Amsterdam, Amsterdam 1098XH, the Netherlands; Consejo Superior de Investigaciones Científicas (CSIC), Instituto de instrumentación para Imagen Molecular (i3M), Universitat Politècnica de València, Valencia 46011, Spain; Institut FEMTO-ST, CNRS UMR 6174, Université de Bourgogne Franche-Comté, Besançon F-25030, France; Department of Biomechanical Engineering, Delft University of Technology, Delft 2628CD, The Netherlands; Department of Robotics and Mechatronics, AGH University of Science and Technology, Krakow 30-059, Poland; Department of Civil, Chemical, Environmental and Materials Engineering, University of Bologna, Bologna 40136, Italy; UMET, UMR 8207, CNRS, Université de Lille, Lille F-59000, France; Instituto de Investigación para la Gestión Integrada de Zonas Costeras, Universitat Politècnica de València, Grau de Gandia 46730, Spain; IMDEA Materials Institute, Getafe, Comunidad de Madrid, Spain; Department of Mechanical Engineering, University of Wisconsin–Madison, Wisconsin–Madison, WI, USA; Hypersmart Matter, AMOLF, Amsterdam 1098XG, The Netherlands; ICREA, Barcelona 08010, Spain; Centre for Metamaterial Research and Innovation, University of Exeter, Exeter EX4 4QL, UK; Graduate School of Engineering, Osaka University, Yamadaoka 2-1, Suita, Osaka 565-0871, Japan; Hokkaido University, Sapporo 060-0808, Japan

**Keywords:** acoustics, additive manufacturing, mechanics, metamaterials, optomechanics, wave dynamics

## Abstract

This broad review summarizes recent advances and “hot” research topics in nanophononics and elastic, acoustic, and mechanical metamaterials based on results presented by the authors at the EUROMECH 610 Colloquium held on April 25–27, 2022 in Benicássim, Spain. The key goal of the colloquium was to highlight important developments in these areas, particularly new results that emerged during the last two years. This work thus presents a “snapshot” of the state-of-the-art of different nanophononics- and metamaterial-related topics rather than a historical view on these subjects, in contrast to a conventional review article. The introduction of basic definitions for each topic is followed by an outline of design strategies for the media under consideration, recently developed analysis and implementation techniques, and discussions of current challenges and promising applications. This review, while not comprehensive, will be helpful especially for early-career researchers, among others, as it offers a broad view of the current state-of-the-art and highlights some unique and flourishing research in the mentioned fields, providing insight into multiple exciting research directions.

## 1 Introduction

Metamaterials—rationally designed composites with properties exceeding those of their constituents—has been a continuously growing field that initially emerged from the study of materials capable of manipulating electromagnetic waves [1], [2], [3]. Metamaterial concepts have rapidly entered and evolved into the domains of acoustics, mechanics, materials science, thermodynamics, condensed matter physics, seismology, civil engineering, and many others. This rapid progress has been driven by advanced characteristics of metamaterials that enable the creation of novel material systems with previously unforeseen functionalities or superior performance attractive in multiple applications, and further stimulated by recent developments in (additive) manufacturing and computational analysis techniques.

The variety of investigated metamaterial configurations, the ranges of their possible functionalities, and the number of existing or potential applications are so substantial that they have become the topic of tens of comprehensive review papers, e.g., Refs. [4], [5], [6], [7], [8], [9], [10], [11], [12], [13], [14], [15], [16], to mention a few. These and other reviews focus on specific nanophononic and metamaterial configurations, properties, or application areas and typically consider relevant developments from a historical point of view. This work has a different scope. It presents a *snapshot* of the research activities in the mentioned fields inspired by lively interactions of the researchers working on various metamaterials topics during the EUROMECH Colloquium “*Emerging topics in acoustic and mechanical metamaterials*” held on 25–27 April 2022 in Benicássim, Spain. This event gathered over 35 seniorand young researchers from different parts of the world and provided a platform for a fruitful exchange of ideas–arguably, the most essential ingredient for advancing research frontiers. This work is aimed at sharing the flourishing collaborative spirit that emerged at the colloquium by overviewing the reported results and summarizing recent advances in nanophononics and metamaterials, thus zooming out from each specific field to get a more general picture. It could be of relevance to all specialists and especially to those who started their research recently and require additional support to gain access to these rapidly developing areas.

The paper is structured by topic. We begin by summarizing the state-of-the-art in nanophononics (Section 2) – the research field focused on developing electro- or optophononic systems and materials that enable the control of vibrations, heat, and quantum communications on the nanoscale [15].

By switching from the nanoscale to the micro-, centimeter- and even meter-scales, we enter the realm of ultrasound, sound, and infrasound elastic or acoustic waves [7] that can be manipulated by means of elastic metamaterials (or “phononic materials”) (Section 3) and acoustic metamaterials (Section 4). Currently, there is no clear common agreement on terminology in the literature on how to differentiate between these two classes of materials. Phononic crystals are typically defined as periodic composites with inhomogeneities that have a high impedance contrast with a base material or as periodic single material systems with nonuniform unit-cell geometries [4, 5, 17, 18]. Acoustic metamaterials can be non-periodic and contain inhomogeneities or nonuniform features of subwavelength sizes causing exotic effective phenomena such as negative bulk modulus and/or negative mass density [4, 10, 19]. Here, for simplicity, we use the term “elastic metamaterial” to refer to phononic materials1In some reviews, the term “phononic materials” is used to describe all categories of architected materials for the manipulation of elastic and/or acoustic waves [8, 16]. in general where waves are admitted in solids and the term “acoustic metamaterials” to exclusively describe media engineered to manipulate acoustic waves in fluids. Therefore, elastic and acoustic metamaterials are differentiated through the properties of the medium supporting the wave propagation, with some analogy to Refs. [8], [9], [10, 12]. Note that some metamaterials can manipulate both elastic waves and airborne sound waves, simultaneously [20, 21].

Finally, we overview several research directions in mechanical metamaterials—tailored composites with unprecedented mechanical characteristics, e.g., auxetic behavior (negative Poisson’s ratio), zero or negative effective volume compressibility, or shape-morphing functionality (Section 5).

For each topic, we consider practically relevant configurations, including bulk materials and metasurfaces, and discuss recent trends in their design, analysis, and manufacturing, and also, when relevant, overview the applications of metastructures formed from metamaterials or metasurfaces. We note that this broad review highlights recent research output of the authors, some related studies, and brief topical reviews needed to introduce relevant concepts. It is not aimed as a comprehensive review, which given the rapid and ongoing progress of the field is not easily covered within a single publication.

## 2 Nanophononics

Phonons forming elastic waves can be considered as powerful low-energy information carriers at the core of modern telecommunication devices. Moreover, phonons are efficient carriers of heat; this offers a functional value beyond electrons, spins, and photons. There has been substantial progress in the study of phonons as heat carriers with relevance to several applications, such as thermoelectrics [22] and heat dissipation [23]. Current research directions also include the investigation of robust-to-disorder focusing and imaging at broadband frequencies [24, 25], and harnessing nonlinear effects to enable phonon localization, breathers, bifurcation, and chaos [26, 27].

Among possible approaches to controlling phonons, the use of thin structures (metasurfaces) and cavity optomechanics are particularly promising, although many challenges remain to be solved. Metasurfaces are subwavelength-thickness structures that allow efficient control and manipulation of reflected and refracted waves by tailoring the geometry of the constituent units. During the last decade, increasing attention on metasurfaces in acoustics [16, 28, 29] and optics [30], [31], [32], in combination with advances in nanofabrication techniques, have stimulated progress in developing structures to control phonons on the micro- and nanoscales [33, 34].

Metasurfaces exploit interactions between surface or Lamb waves and locally-resonant phononic units. For a metasurface composed of a one-dimensional (1D) array of cylindrical pillars, for example, the graded variation in the pillar height enables sub-wavelength focusing that is robust with respect to disorder in geometric parameters or in frequency fluctuations [24]. The phase shift of the transmission coefficient associated with each resonance of the pillars (e.g., the first or the second bending and compressional modes) is equal to *π*. In order to achieve a phase shift that spans a range of 2*π*, as required for a metasurface, an essential feature of the design is to superimpose two resonances at the same frequency. In our case, the second bending and the first compressional resonance frequencies are made degenerate by tailoring the geometrical parameters. Then, the transmission phase shift of the different units in the metasurface is made to span a range of 2*π* by gradually varying the height of the pillars as a function of position. For example, silicon pillars that are 120 μm in diameter and with a lattice pitch of 150 μm can focus the flexural plate mode propagating in a silicon (crystalline semiconductor) plate at 8.05 MHz, which corresponds to about a three times larger wavelength than the diameter of the pillars [24]. The sub-wavelength focusing is achieved as far as the parameter *F*/*D* remains below 0.25, where *F* is the focal length and *D* the length of the metasurface [24]. An experimental demonstration of focusing functionalities has been obtained by using 3D-printed polymer materials in the mm range [25]. Similar functionality can be achieved by using elliptical pillars that have two degrees of freedom, to implement the gradual change in phase shifts [35] while avoiding the difficulty of height variation in a microfabrication process. Although the design of metasurfaces based on elliptic scatterers has been already proposed in optics [36, 37], its application to the manipulation of elastic Lamb waves in a plate is new.

Alternatively, resonator-to-resonator coupling in dual resonators or in a periodic nanostrip can be exploited to achieve coherent driving of the mechanical micro- or nano-resonators with surface waves and, reciprocally, to control surface wave propagation at a deep sub-wavelength scale. The confinement and enhancement of elastic energy in volumes much smaller than the excitation wavelength can be demonstrated by using either individually fabricated pillars by focused ion beam induced deposition (FIBID) or periodic high-aspect-ratio nanostrips, both integrated on a highly coupled piezoelectric substrate [26, 27]. In the case of dual resonators, optical measurements by laser scanning interferometry have been used to enable direct observations of the frequency splitting response and dipole-like mode shapes of the coupled resonators. The gap distance and the orientation of the coupled resonators with respect to the surface acoustic wave (SAW) wavevector highlight the vectorial nature arising from the dipole-like mode shape. In the case of nanostrip resonators, classical electroacoustic impedance measurements can be achieved at a different stimulation power. The elastic field behavior can be further controlled through other types of coupling by reducing the resonator gap and the discrete resonator mode. For instance, nonlinear geometry as well as elastic nonlinearity can be achieved through the control of geometrical parameters of the unit cell and the resonance mode of a single nanostrip finger, leading to a variety of nonlinear interaction schemes. In the case of a compressional mode of the nanostrip, reducing the gap distance and increasing the SAW amplitude induces a frequency shift of the device and allows the implementation of high-frequency phononic-NEMS (nanoelectromechanical systems) circuits with complex dynamics.

Despite the reported progress, studying phonons on their own remains a challenge. A possible solution to overcome this hurdle implies the use of optomechanics (OM) concepts. OM exploits the confinement and interaction of optical and mechanical waves. OM interaction forms a foundation for the relationship between phonons, considered as mechanical modes, and photons, which results in the transduction of optical modes in the THz into mechanical modes in the sub-GHz regime and vice versa. An alternative is the transduction of electrically driven mechanical energy from, e.g., SAWs into an optical or electronic signal.

Common realizations of OM structures, also known as phoXonic crystals [38], rely on cavity optomechanics that enables coupling of electromagnetic radiation and mechanical vibrations enhanced by confining the electromagnetic radiation in a cavity. The main attention so far has been on studying 1D cavities realized both in silicon-on-insulator (SOI) [39] and in nanocrystalline silicon (nc-Si) membranes [40] at room temperature and ambient conditions. The mechanical Q factors at 2.4 GHz in the resonators fabricated in nc-Si were found to be higher compared to those structures fabricated in SOI, 745 compared to 1680 in SOI and nc-Si, respectively, which led to the question as to the origin of the reduced losses, or higher Q, in nc-Si OM cavities in what otherwise were identically geometrically designed 1D OM crystals. The study of nc-Si with three different average grain sizes showed that higher thermal diffusivity or more efficient heat transport was associated with the larger mean nc-Si size (290 nm) or the larger volume ratio of nc-Si volume to that of grain boundaries, supported by heat dissipation rates being the largest for the 290 nm diameter nanocrystallites. OM crystals and nc-Si membranes have promising potential for sensing, communication, and coherent quantum control of mechanical oscillators [41, 42].

**Figure 1:**
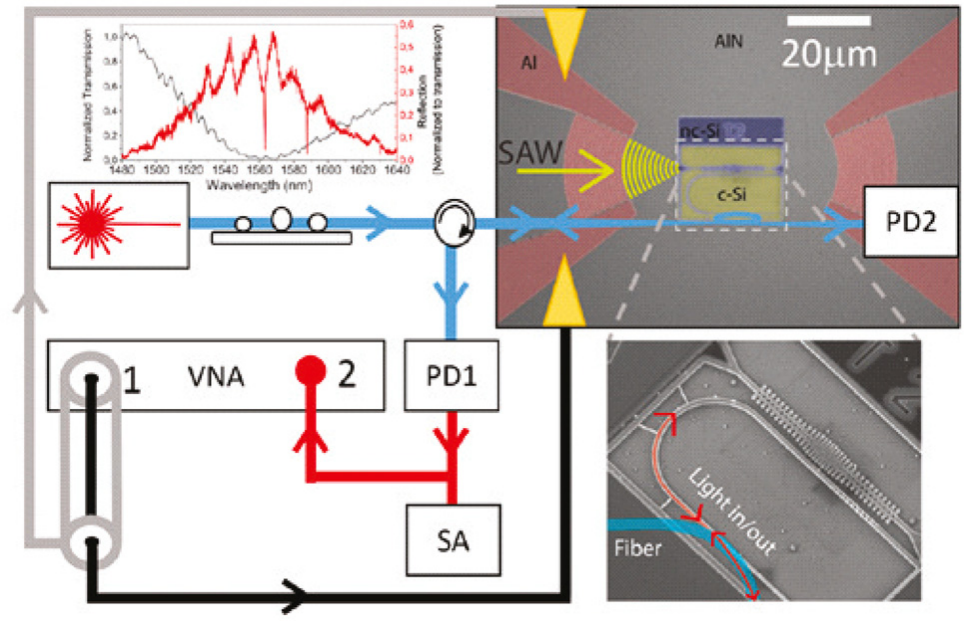
The NOEMS platform for room temperature operation at 2 GHz. The top left inset shows the normalized transmission (black line) and normalized reflection (red line). Reprinted with permission from D. Navarro-Urrios, M. F. Colombano, G. Arregui, et al. *ACS Photonics*, 2022, 9, 2, 413–419. Copyright 2022 American Chemical Society*.*

One of the “hottest” research directions in this field is to realize nano-opto-electromechanical systems (NOEMS) enabling the synergistic coexistence of electrical, mechanical, and optical signals on a chip. The major challenge here is to make such technology platforms fully compatible with mainstream CMOS technology. The quest to demonstrate an OM circuit based on nc-Si OM resulted in an on-chip NOEMS platform at the proof-of-concept stage, as shown in Figure 1. This is an electrically pumped OM nanobeam by 2 GHz surface waves in a platform based on nc-Si, Si, and AlN with metal contacts that can successfully convert microwave signals into optical signals [43]. In these experiments, the achieved mechanical Q factor is 4000, limited by losses in the material and the optical Q factor of 10,000 at room temperature. The data analysis yielded 200 coherent phonons per microWatt, and a peak sensitivity of below three phonons, which is very promising for a platform fully compatible with Si technology. The performance of this and similar devices critically relies on efficient control of thermal energy transfer and thermal conductivity. The analysis of the impact of grain boundaries on thermal transport in silicon micro- and nano-structures [44] clarified that the crystalline state is a key parameter in NEMS and/or NOEMS. Similar devices exploit non-linear physical mechanisms, such as the chaos regime [45] and synchronization [46], to unveil rich sets of fundamentally different complex dynamics and to switch on/off synchronization of the mechanical dynamics in OM crystal cavities. These developments open new horizons for exploiting silicon-based OM crystals in neurocomputational networks and chaos-based applications.

The extension to two-dimensional (2D) optomechanical platforms enables the realization of Anderson localization of optical modes by using disorder in cavity optomechanics [47]. For instance, consider a waveguide etched in a suspended silicon membrane with a slotted line air defect. The air slot allows strong confinement of the electromagnetic field and enhances the ability for light to couple to in-plane mechanical motion, while inherent and unavoidable fabrication imperfections are adequate to induce sufficient back-scattering. The resulting tightly confined Anderson-localized modes can be driven to enable mechanical amplification and self-sustained phonon lasing via optomechanical back-action. This opens perspectives for studying multiple scattering and Anderson localization of bosonic excitations with parametric coupling to mechanical degrees of freedom.

It is worth remembering that open nanophononic structures with resonators suffer from radiation loss that results in a finite lifetime of the modes. Instead of normal modes with pure real eigenfrequencies typical for closed systems, open systems possess quasi-normal modes (QNMs) with complex-valued eigenfrequencies and diverging power at infinity. QNMs have been widely discussed in photonics [48, 49], but the advanced tools required for their analysis of acoustic and elastic waves still require improvement [50]. Of special interest is the determination of complex eigenvalues and eigenmodes, and the definition of an adequate modal volume and the elastic equivalent of the Purcell effect [51]. A practical way to obtain all QNMs of an elastic resonating structure is to rely on a stochastic method to evaluate all possible resonances from the response of a system to a random force [52]. Because the total energy of QNMs is unbounded, the usual normalization relations for normal modes are not applicable, while a multipole expansion with complex coefficients can be obtained, and a complex modal volume can be defined.

Finally, a promising direction to prevent and minimize phonon dissipation and losses within the GHz range is to engineer the geometry of the nanostructure in order to open wide frequency gaps in the spectrum inducing destructive interference in all directions [53]. The related platform, demonstrated only very recently, enables the creation of waveguides and cavities where the OM coupling can be exploited to activate nonlinear dynamics and coherent amplification of vibrations at room temperature [54].

## 3 Elastic metamaterials

The field of elastic metamaterials has largely extended in the last few decades from studying mass-spring systems [55, 56] and circular periodic heterogeneities or nonuniform features [5, 17, 18] to developing intricate designs with spiral patterns [57], hierarchical [58], or bio-inspired organization [59, 60]; from the analysis of Bragg scattering and local resonances of elastic waves [19, 61] to topological optimization of band structures [62], and studying tunable [63], nonlinear [64], and time-varying metamaterials [65]; from broadband wave attenuation [5] to futuristic applications including speech recognition [66], active thermal cloaking [67], realistic energy harvesting [68], and seismic wave mitigation [69] systems. This progress has become possible thanks to advances in computational techniques and manufacturing approaches that enable the analysis, optimization, and production of complex metastructures at various size scales.

In the following, we summarize recent trends in the design (Section 3.1) and analysis (Section 3.3) of elastic metamaterials as well as in the studies of nonlinear, time-varying, and externally controlled metamaterial media (Section 3.2). Finally, we consider several emerging applications for elastic metastructures (Section 3.4).

### 3.1 Design of elastic metamaterials

Most of the fascinating functionalities of elastic metamaterials rely on their ability to generate absolute (directional) band gaps-frequency ranges with inhibited (directional) wave propagation.

Band-gap engineering and, on a broader scale, control over wave dispersion, are implemented by designing the structural topology of a metamaterial that can be done based on intuition or bio-inspiration, by means of phenomenological approaches or computational optimization, or any combinations of these. Such strategies extend possible phononic designs and further expand the range of elastic metamaterial functionalities or possible devices. Below, we present some examples of recent design ideas with a focus on lightweight configurations that can suppress low-frequency elastic waves. Such structures are vitally important for diverse applications that require vibration isolation and wave manipulation. Furthermore, recent design preferences have been shifting towards single-material configurations to facilitate manufacturing.

Elastic metamaterials can be formally divided into 1D (metabeams or metarods), 2D (metasurfaces), and 3D (bulk) configurations according to the directionality of their wave attenuation performance.

**Figure 2:**
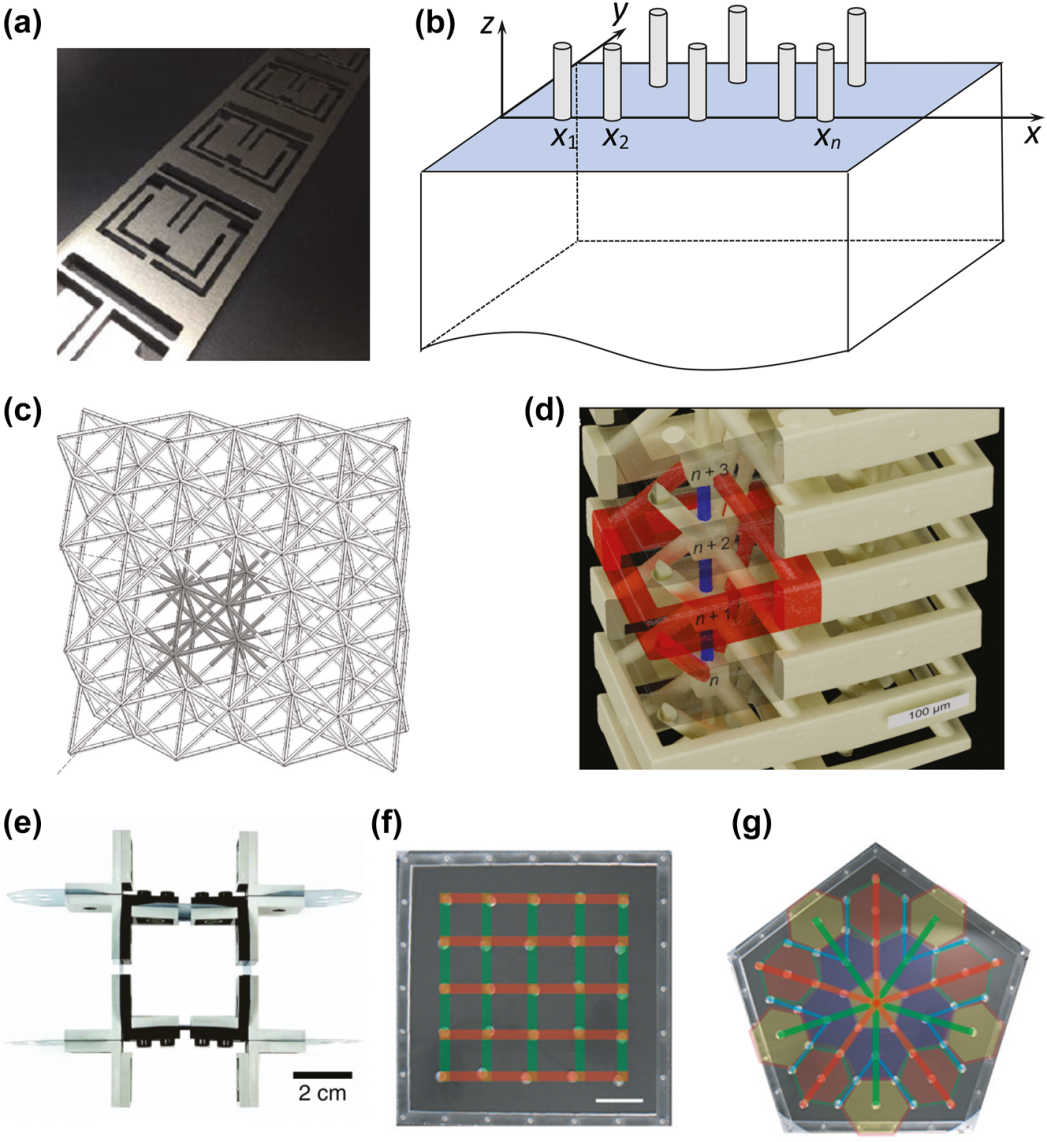
Examples of phononic materials. (a) Meta-beam with quadruple-mode resonators (adapted from Figure 1 published in: Kentaro Fujita; Motonobu Tomoda; Oliver B. Wright; Osamu Matsuda; *Appl. Phys. Lett.* 115, 081905 (2019), Copyright © 2019); (b) metasurface with aperiodic resonators; (c) octet panel with a highlighted unit cell; (d) 3D phononic material with roton-like dispersion (originally published in Ref. [86]); (e) flexible metamaterial with a “rotating-square” structure (adapted from: B. Deng, P. Wang, Q. He, V. Tournat, K. Bertoldi, Metamaterials with amplitude gaps for elastic solitons, *Nat. Commun., Nature Research*, 2018); (f–g) self-assembled lattices within 2D magnetic boundaries with square and quasi-crystal configurations.

An interesting example of an intuition-based 1D design is an aluminum meta-beam with periodic quadruple-mode resonators that exhibits a band gap around 1 kHz (Figure 2a). This structure can efficiently dampen out compressional, in-plane shear, flexural, and torsional vibrations, as proven experimentally [70]. A tapered version of this structure has also been demonstrated with a wide-band response [71]. Another example is a bio-inspired meta-rod with a structure resembling that of *Turritella Terebra* shell. 3D-printed prototypes reveal complex resonating modes determined through resonant ultrasound spectroscopy and are in good agreement with finite-element simulations (unpublished work).

Elastic metasurfaces – structured materials, typically, equipped with mechanical resonators – have been opening bright possibilities for the control of surface elastic waves across different frequency ranges [16]. Hybridization between propagating surface waves and localized resonator motion enables probing the wave dispersion [72] and brings rise to peculiar phenomena such as surface-to-bulk wave conversion [73, 74], rainbow trapping [75], wave localization [76], and non-reciprocal propagation [77]. Typical metasurfaces consist of a cluster of oscillators arranged in a periodic fashion over an elastic half-space. A new emerging trend is, rather, the development of aperiodic metasurfaces (Figure 2b). The work of Marchal et al. [78] provides experimental data on the dynamics of confinement of elastic waves within a cavity in a phononic crystal. A noncontact laser ultrasonic technique is used to monitor *in situ* the displacement field within the cavity as a function of time and space after a surface acoustic pulse has been excited outside the cavity. It is shown that the time evolution of confinement is distinct according to the symmetry of the eigenmode. More recently, the same team has investigated the localization of surface waves after propagation in a diffusive medium consisting of pillar-shaped mechanical resonators randomly distributed on a surface (unpublished ongoing work). The samples are formed by an assembly of several thousand aluminum pillars arranged randomly on a 200 nm thick film, also made of aluminum, deposited on a semi-infinite silica substrate. Both the diameter and the height of the pillars are 300 nm. Depending on the samples, the filling ratio (ratio of the total section of the pillars to the surface of the sample) is between 7 and 9%. The localization of elastic energy in such a random structure results from the interference of the waves bouncing back and forth within the free surface surrounded by pillars. For the surface waves with a spectral content extending up to 2 GHz, the measurements enabled the reconstruction of the cartography of surface displacements in a purpose-built phononic cavity that clearly shows stationary waves, which in this particular example last for approximately 2.5 ns.

In the realm of 3D elastic metamaterials, representative examples are frame-based lattices and shell-like structures known for their exotic ability to manipulate waves [79]. The octet-like lattices (Figure 2c), among others, have a broadband gap, in which local-resonance and Bragg scattering naturally coalesce [80]. Shell-based structures (of Schwartz primitive type) with topology based on periodic minimal surfaces also have a promising potential for wave control. By changing the geometrical parameters of the lattices, one can easily tune the band structure and achieve wave focusing [81], thus laying the ground for the development of novel wave control structures for vibration isolation, focusing, and, possibly, energy harvesting.

Bio-inspiration and structural hierarchy can provide powerful tools to design lightweight elastic metamaterials [82]. Inspiration can be drawn from numerous examples of natural structures providing efficient vibration attenuation and wave control. Bio-inspired designs have shown interesting wave attenuation properties in hierarchical (diatom-like) [58], spiral-shaped (cochlea or shell-like), and frame (spider-web-like) elastic metamaterials [59, 60] (Figure 2c). Band gaps relative to the corresponding non-hierarchical structures are mostly preserved in both types of structures, but additional hierarchically induced band gaps appear in some cases. Furthermore, hierarchical configurations are amenable to a high degree of tunability using a limited number of geometrical parameters, and simultaneously ensure reduced structural weight. The role of viscoelasticity, which is essential for complex designs, was also considered [58, 83, 84].

A strong source of inspiration for creating new elastic meta-designs remains solid-state physics. For instance, the research group of M. Wegener has proposed a 3D roton elastic metamaterial for manipulating transversal and longitudinal elastic waves (Figure 2d), that exhibits low-frequency dispersion similar to the famous roton dispersion, which was suggested by Landau around 80 years ago to explain the sharp transition of specific heat in liquid Helium-4 observed at a critical low temperature [85]. The term “roton” originates from the dependence of the phonon energy on the momentum. For small momenta, the phonon energy reveals a linear dependence, while a local minimum of energy versus momentum occurs for larger momenta. To achieve a similar behavior for elastic waves, it was proposed to introduce beyond-nearest-neighbor (i.e., non-local) interactions between effective masses that must dominate over nearest-neighbor interactions. This idea can be realized in a practical 3D elastic metastructure by connecting small cubes, which act as effective masses, by means of cylindrical rods, which act as effective springs. The structure is embedded in an auxiliary frame whose aim is to mediate the beyond-nearest-neighbor interactions, realized from a single polymer by 3D additive manufacturing [86]. The designed metamaterials were tested experimentally and revealed the roton dispersion for a transversal elastic wave at ultrasonic frequencies in micrometer-sized samples [86].

Another prominent example of a physics-inspired elastic metastructure is a mechanical analog of twisted bilayer graphene [87, 88]. This is a structure composed of two graphene monolayers placed in direct contact with each other after rotating one of them by a certain small angle *θ*. This angle is responsible for forming a so-called Moiré pattern – a periodic arrangement that could alter the electronic band of the bilayer graphene. At a specific “magic” interlayer rotation angle, twisted bilayer graphene develops quasi-flat bands through an unconventional mechanism connected to carrier chirality, which enables a wealth of exotic, correlated-electron phases in the system [89]. The corresponding mechanical realization is represented by two vibrating plates patterned with a honeycomb mesh of masses and coupled across a continuum elastic medium. By connecting the movement of the upper plate to the movement of the bottom plate by means of an appropriate coupling coefficient, it is shown that flexural waves can exhibit vanishing group velocity and quasi-flat bands at magic angles are in close correspondence with electrons in graphene models [87]. The strong similarities of spectral structure and spatial eigenmodes in the two systems demonstrate the chiral nature of the mechanical flat bands [88]. Experimental realization of this proposal is also in view [88].

Finally, an alternative design approach aimed at optimized elastic metamaterials in terms of target characteristics (e.g., the band-gap width) is based on computational tools. This approach has proven to be fundamental for exploring the vast geometry-property spectrum [90], [91], [92], [93], [94]. Yet, critical steps in the optimization procedure are often conducted manually. For example, to optimize a metamaterial waveguide for wave attenuation and appropriate mechanical performance, one needs first to analyze the band structure of a representative unit cell and adjust the required frequency range. Several configurations may then be tested in terms of wave transmission and maximum stress induced by external loading. Next, the best design is selected for further optimization, whereby the design space is surveyed (also performing sensitivity analysis) in pursuit of an optimized configuration. The final design can then be fabricated and validated experimentally. In contrast, it is more desirable to design such structures systematically, e.g., by means of topology optimization – a technique that combines finite element analysis with gradient-based optimization. One of the recent realizations of a topology optimization procedure combines such enriched finite-element formulation in the analysis with a level-set function that is used to represent topology [95, 96]. Such computational tools can effectively be used to design multi-functional phononic media that exhibit, e.g., band gaps and required mechanical performance simultaneously.

### 3.2 Time-varying, nonlinear, and tunable elastic metamaterials

In parallel to the search for new elastic meta-configurations, substantial attention has been given to harnessing non-trivial material behavior to generate or enlarge band gaps and to break the limits imposed by linear base materials on controlling wave dispersion. One of the recent examples to extend band-gap tunability by preserving the geometric structure is to use 3D-printed photo-responsive polymers, which can reversibly change their Young’s modulus by up to 30% upon laser illumination [97].

Nonlinear phenomena are another area that continues to attract active research within the field. Material nonlinearities may be activated by exciting large-amplitude mechanical waves in highly compliant elastic meta-media, resulting in a separate class of elastic metamaterials called “flexible mechanical metamaterials” [98]. While the static character of such structures has been widely studied, elucidating their dynamic properties is still in its early stages, especially with regard to nonlinear dynamics. The nonlinear properties can be designed in a rational way, using appropriate lump-parameter models and analytical developments [98], [99], [100], to enable control of large-amplitude elastic waves. A prototype of a flexible mechanical metamaterial that has been most studied in the context of nonlinear waves is the “rotating square” structure [98]. It consists of rigid square masses, connected at their corners to neighboring masses by thin elastic ligaments (Figure 2e). Several degrees of freedom in displacement and rotation must be considered for each rigid unit and the thin elastic ligaments can be modeled by longitudinal, shear, and bending springs. The discrete equations of motion obtained with this lump-parameter model serve as the basis for the numerical simulation of nonlinear wave propagation. Also, using the appropriate assumptions on these discrete equations of motion for the wave effect under consideration, analytical solutions can be found in most cases. In particular, it has been shown that rotating square structures can support vector elastic solitons – large-amplitude pulses propagating without changing shape due to the perfect balance between nonlinear and dispersive effects [99, 100]. The coupling between rotational and translational degrees of freedom gives the vector character to this solitary wave. Such vector solitary waves in a flexible mechanical metamaterial exist theoretically and experimentally for a particular range of amplitudes that can be tuned by the specific design of the metamaterial, which highlights “amplitude gaps” for vector elastic solitons, by analogy with frequency gaps in dispersive linear media [100]. Various wave or material configurations in this context have been studied [101], [102], [103], [104], among which is a “mechanical diode” for vector elastic solitons [100]. In the case of multi-stable designs, so-called transition waves, or topological solitons, can propagate. These waves sequentially switch the units of the system from one stable state to another, resulting in the reconfiguration of part or all of the medium [105], [106], [107].

In conventional solid structures, such as rods and beams, nonlinear effects may be introduced through the stress-strain relation and/or the strain-displacement gradient relation. For a homogeneous medium, nonlinear dispersion relations may be obtained in exact and analytical form as shown for rods [108, 109] and beams [108, 110]. In these works, a theory is given that provides the nonlinear dispersion relations without resorting to perturbation methods, thus being applicable to strongly nonlinear models. In Ref. [109], a harmonic dispersion relation for the prediction of the spectra of the generated harmonics is also provided. This general approach has also been combined with the standard transfer-matrix method to obtain approximate nonlinear dispersion relations for periodic media. One can consider, for example, longitudinal waves in a continuous thin rod with a periodic arrangement of resonators [111] or material properties [112]. This allows for the analytical prediction of band-gap characteristics. Among the interesting results, for example, is a demonstration that large deformation in a nonlinear elastic metamaterial may cause a pair of isolated Bragg-scattering and local-resonance band gaps to coalesce to create a much larger band gap [111].

An alternative approach for elastic metamaterials with non-trivial material behavior is time-varying metamaterials, in which time acts as a new degree of freedom [113]. The concept of time symmetry was first violated by temporal modulations in photonic materials. Since then, this phenomenon has attracted much interest leading to the implementation of novel nonreciprocal wave management techniques [114]. Moreover, the need for a fundamental understanding of wave phenomena in the time domain has led to the discovery of other surprising physical effects, such as frequency conversion [115], temporal aiming [116], or an inverse prism [117]. The properties of time-varying metamaterials can change in time due to temporal variations in some of their physical parameters induced by an external energy source, thus changing their response over time, which can be considered as next-generation metamaterials [118]. A basic time-varying metamaterial can be represented by a temporal multi-layer scheme of an unbounded string, without dissipation, consisting of a cascade of different speed media over time. An initially transverse wave propagating in such a string splits in multiple forward and backward waves at each time interface, due to the change of speed; this complicates the wave dispersion analysis as further discussed in Section 3.3.

In a more general definition, metamaterials that exhibit spatial and temporal changes are called spatiotemporal metamaterials. Despite these metamaterials being proposed many years ago [119], this topic was hardly explored for a long time due to difficulties in the practical implementation of this concept. The recent renewed interest in spatiotemporal metamaterials is due to the spectacular developments in new technologies and artificial intelligence, which promise a bright future for this new generation of metamaterials [120], [121], [122], [123], [124]. Although this branch of metamaterials is still very young, studies combining Floquet engineering and metamaterials show great possibilities for the development of interesting applications based on the control of the temporal properties of waves. Time-varying media are promising candidates for controlling wave frequency without resorting to nonlinear effects, which pose theoretical and practical difficulties.

On the other side, one of the major limitations of linear and nonlinear elastic and acoustic metastructures is their fixed topology which results in predefined wave dispersion once these structures are fabricated. Despite many theoretical and computational studies on tunable metamaterial configurations, the practical realization of experimental platforms remains limited because of challenges in manufacturability, the destructive nature of reprogramming, and inherent nonlinearities [125], [126], [127]. A valid path for functional tunability of metamaterial matter is external stimuli, e.g., either static or dynamic load, which can effectively induce an external field to tune the material. This, however, can translate to large shape change in the material with the potential rise of instabilities, phase transitions, degradation, and geometric frustration. This can hinder our ability to utilize programmable materials [107, 128]. To overcome these limitations, the group led by O. R. Bilal has proposed to obtain programmable dynamical characteristics by utilizing tunable nonlinear magnetic lattices [129], [130], [131], [132]. Such lattices can be created by confining self-aligned, free-floating disks of embedded permanent magnets in a range of symmetries within a fixed boundary and a pre-engineered magnetic field (Figure 2f–g). The magnetic repulsive forces between the boundary magnets and the free-floating disks cause the disks to self-align and rest within their surrounding magnetic potential wells. Each disk or group of disks can act as a single building block of the lattice. These blocks repeat in space, forming a 1D or 2D configuration with unique wave propagation characteristics. The geometry of the confining boundary, disk symmetry, and the number of embedded magnets within each disk can be designed such that the tuning of the boundary’s magnetic field controls the assembly of the disks. The relative positions and orientation angles between the disks define the corresponding metamaterial phase (ordered, quasi-ordered, or random) and its stability conditions (mono-stable or multi-stable). By changing the boundary geometry, the number of disks, their symmetry, and therefore the resulting magnetic couplings within the unit cell, the transmittable frequencies through the metamaterial can be programmed. The proposed platform can be utilized to engineer self-assembled meta-structures capable of manipulating low-frequency waves, within a relatively small volume, while utilizing negligible mass. Moreover, the nonlinear potentials between the disks and their boundaries can be harnessed to demonstrate phenomena with nonlinear parallel such as amplitude-dependent response, bifurcation, chaos, non-reciprocity, and solitons. In addition, the self-assembling nature of the disks can be key in creating reprogrammable materials with exceptional properties.

### 3.3 Analysis techniques for elastic metamaterials

Complex metamaterial designs, material or geometric nonlinearity, and strong external stimuli require efficient analysis techniques that can deliver reliable results and useful insights into the physical aspects of the wave propagation process.

Currently available commercial finite-element software allows exploring almost arbitrarily complex designs of 3D elastic meta-media. The analysis steps and related details remain, however, the responsibility of the software users. For instance, it is important to verify the consistency of the choice of a unit cell and the first irreducible Brillouin zone to exclude the possibility of erroneous conclusions. This is especially relevant for lattices of a low-symmetry group and when base material(s) behaves non-linearly or non-elastically [133]. There are two possible shortcomings here: (i) the use of non-primitive unit cells for the analysis of directional band gaps and (ii) the limitation of the computations to the reduced Brillouin zone paths. These can influence the accuracy of band-gap estimations and result in fictitious bands. The latter numerical phenomenon is known as spatial aliasing [134]. Low-symmetry designs can be analyzed by using a recently proposed algorithm to construct an irreducible Brillouin zone for any crystal structure in 2D and 3D [135].

In the realm of metasurfaces, finite-size structures with aperiodic clusters of resonators are nowadays analyzed numerically. However, it is possible to develop an analytical framework based on the multiple scattering technique to model the wave field in elastic metasurfaces [87]. To this purpose, one can utilize the solution of the classical Lamb’s problem as a Green’s function to describe the multiple scattered fields generated by a cluster of mechanical resonators attached to a surface. Using this approach, Refs. [76, 136, 137] discuss the interplay between surface waves and resonators in both 2D and 3D contexts by analysing a series of metasurface configurations for wave conversion, wave trapping, and topological protection of surface edge states.

Nonlinear and time-modulated metasurfaces require more advanced modeling strategies [77, 138]. For time-modulated scenarios, a metasurface can be composed of resonators with a periodic space-time modulation of stiffness. By means of an asymptotic approach, one can obtain an analytical solution describing the dispersion properties of such a structure. The dispersion analysis shows that a spatiotemporal stiffness modulation yields non-reciprocal features in the Rayleigh wave spectrum where the frequency content of a propagating signal can be filtered and converted to bulk waves [77].

A nonlinear metasurface with an array of Duffing oscillators, namely spring-mass resonators featuring a nonlinear force-amplitude response of a cubic type, can be studied by adopting a leading-order effective medium description. This approach allows the prediction of the amplitude-dependent dispersive characteristics of Rayleigh waves. It was shown that hardening nonlinearity shifts and reduces the attenuation bandwidth of the metasurface, while softening nonlinearity, in contrast, yields lower and broader frequency gaps [138]. This opens new opportunities to realize tunable devices for surface wave control.

Nonlinear metaclusters and metasurfaces with wave scatterers displaying nonlinear properties can, alternatively, be analyzed by perturbation theory. This approach is illustrated in Ref. [139] for a nonlinear multiple scattering system represented by a beam with a cluster of scatterers having a cubic dependency of the attachment forces on the displacement. The obtained solution shows that both reflected and transmitted amplitudes depend on the direction of incidence. This implies that apart from asymmetric reflection, possible with linear scatterers, a cluster of nonlinear scatterers may also present asymmetric transmission, thus breaking reciprocity. Further, the scattering properties of the system depend on the incident wave amplitude. Consequently, the tunable asymmetric scattering properties can be used to construct acoustic devices, e.g., tunable filters or frequency converters [139]. Such a frequency converter, which – when hit by a wave of certain frequency – transmits only waves at the third harmonic can be built with a cluster of three scatterers. In the cluster, the two outer scatterers are linear and are designed to prevent transmission of the fundamental frequency and the reflection of the third harmonic. The middle scatterer is cubically nonlinear and thus generates a third harmonic wave. A significant portion of the incident wave is then reflected, while the transmitted fundamental-frequency amplitude and the reflected third-harmonic amplitude both vanish at the nominal working frequency, regardless of the incident amplitude. At the same incident frequency, the cluster transmits the third-harmonic wave. Therefore, the cluster acts as a frequency converter, where only the third-harmonic component is transmitted. This frequency converter also demonstrates the breaking of reciprocity, where incidence from one side, at a specific frequency, results in transmission of the third harmonic component, whereas incidence from the other side results in no transmission [139]. Therefore, the generation of higher-harmonic waves and the correction to the fundamental frequency wave can lead to some exceptional scattering behavior when a number of scatterers are clustered on a beam. Such systems may facilitate the implementation of elastic analogs of electronic devices, such as isolators and logic devices.

### 3.4 Applications of elastic metamaterials

Existing and emerging elastic metamaterials have a promising potential in multiple applications that require vibration mitigation and waveguiding, wave focusing and collimation, wave lensing and demultiplexing, sensing and scattering-free transmission, energy conversion, acoustic cloaking, topology-protected states, etc. Elastic metasurfaces open new possibilities to design and realize similar devices for surface waves. Many of these applications are mentioned above or thoroughly discussed elsewhere [5, 8, 16, 140], [141], [142]. Here we consider several recent developments relative to elastic metamaterials for seismic wave mitigation and energy harvesting and highlight two more exotic application areas – active cloaking and speech recognition.

A new twist to *seismic phononic media* [143, 144] is given by bio-inspired seismic insulators [69]. These are formed by periodic arrays of building blocks that replicate the mechanics of the human body and/or animal locomotion. Such blocks are composed of linkages mimicking human bones and stretchable cables replicating muscle tendons. The working mechanism is based on the biomechanics of animals that can adjust their natural vibration frequencies to reach a state of resonance with the forces produced by the contraction of muscles during locomotion. This frequency tuning process enables the production of motion with low energy consumption [145]. Similarly, human bones in legs and arms behave as pendulum systems that are bent by muscles to tune their resonant frequency, while the tendons act like nonlinear springs and shock absorbers. The proposed meta-isolators work in an opposite fashion: they tune the nonlinear stiffness of the tendons in order to avoid resonance with leading earthquake frequencies. The effective properties of such devices (e.g., the effective vibration period and the effective damping coefficient) can be tuned by changing the geometry of a device, the friction coefficient of the sliders, the preconditioning, the energy dissipation capacity of the tendons, the ratio between the design value of the re-centering force, and the maximum vertical load [69]. An additional advantage is that the fabrication does not require expensive materials and is partially or fully achievable with ordinary 3D printers. The metallic parts can be manufactured using standard lathe machines and/or fabricated with desktop metallic 3D printers. Actively controllable versions of such metamaterials can be equipped with actuated cables to provide supplementary re-centering capacity and stiffness control. The latter is useful to adjust in real-time the fundamental vibration period of the device to the earthquake frequency and energy content, as well as to re-center the system at the end of a seismic oscillation in case of permanent deformations. These metastructures pave the way to a customized approach to protect artworks, houses, and essential equipment in industrialized and developing countries [143, 146].

*Vibration energy harvesting* (VEH), i.e., the conversion of mechanical vibrations into electrical energy, is another popular application area of elastic metamaterials, which has approached the proof-of-concept stage [147]. Elastic metamaterials are promising to address a current challenge in the field – to develop microscale harvesters in the form of micro-electromechanical systems (MEMS). The implementation of VEH solutions for efficient energy conversion on the microscale to meet the decreasing power roadmap of microelectronics within 5–10 years is now recognized as a crucial goal [148]. To reach this goal, recently developed elastic metastructures with endowed piezoelectric inserts rely on the idea of creating a parabolic acoustic mirror, introducing point defects in periodic lattices, or an acoustic lens to concentrate narrow-band vibrations [149]. An alternative approach proposes to exploit graded arrays of sub-wavelength resonators [150].

The basic design can be represented by local resonators on waveguides in the form of beams to create a meta-frame or meta-lattice controlling the propagation of elastic waves. This idea can be extended to 3D architectures, as has been done in [151] to achieve selective filtering of transverse and longitudinal waves. Here, however, the focus is on planar structures, in order to keep compatibility with the typical industrial processes adopted in industrial MEMS manufacturing. The actual realization of the described metastructure includes square unit cells of a chiral structure and suitably graded local resonators at the corners. The interplay between chirality, local resonance, and grading enables the achievement of a continuous redirection of the elastic waves, which are finally confined and amplified inside the square unit cell. By implementing the piezoelectric coupling in some specific target resonators, the meta-lattice can be tailored for energy harvesting applications, minimizing energy losses due to wave scattering. The performance of the meta-frame is preliminarily checked numerically. The constituents of the unit cell are modeled using Timoshenko (shear flexible) beams with six degrees of freedom per node to reduce the high computational burden that would arise when using 3D solid elements. The dynamic response to narrow-band signals confirms that elastic waves are effectively redirected and strongly interact with the resonators equipped with a piezoelectric material, with the consequence of enhanced energy harvesting with respect to conventional single-resonator systems. These results pave the way for the development of microscale devices by means of standard etching processes, with the deposition of lead-free piezoelectric materials such as aluminum nitride. The microscale prototypes are characterized by means of advanced techniques, making use of ad-hoc excitation probes and specific measurement procedures [68].

*Cloaking* represents one of the unusual functionalities of elastic and acoustic metamaterials, which cannot be achieved by conventional engineering materials. Recent developments in thermal cloaking involve using active sources to cloak objects from thermal measurements instead of exotic materials. Rather than working with the heat equation in the time domain as in [152], the proposed method relies on the frequency domain formulation for Helmholtz equation solutions with complex wavenumbers, thus, extending the active exterior cloaking for the Helmholtz equation method [153] from positive wavenumbers to complex ones. The convergence estimates for this cloaking approach are given in [154]. This analytical approach allows both gaining a deeper insight into the physical problem and establishing useful estimates regarding the cloaking efficiency in both time (for the parabolic heat equation) and frequency (for the Helmholtz equation) domains. A similar approach can be developed for cloaking in dispersive media.

The current keen interest of the metamaterial community in the active exterior cloaking theory for the parabolic heat equation [152] is illustrated by a fully numerical optimization route [67] that stimulates practical realizations. Yet, actual production and use of such and other devices at the nanoscale requires low-cost fabrication routes to cut manufacturing expenses. For this purpose, a promising solution is the soft nanoimprinting lithography technique that provides an exciting opportunity for the fabrication of nanostructures in a scalable, fast, and inexpensive way. It uses pre-patterned soft elastomeric stamps to induce a nanostructure onto a variety of materials such as conductive polymers [155] and cellulose [156]. Patterned stamps can also induce the long-range ordering of metal colloids [157, 158] and perovskite nanocrystals [159] in what is known as template-induced self-assembly. In all cases, the resulting architectures can exhibit a resolution below 100 nm while covering an area of 1 cm^2^. This fabrication route allows the combination of the desired properties of a pattern with those of the original material, resulting in a new generation of inexpensive components such as biodegradable photonic films, highly efficient SERS platforms for sensing, chiral metamaterials, improved efficiency solar cells, and more.

An exciting newly emerging application domain for mechanical metamaterials is *computation* and *artificial intelligence*. Examples of intelligent tasks that have been theoretically or experimentally demonstrated in metamaterials include the error-correction of communication signals [160, 161], implementing digital computations [162, 163] and finite-state machines [164], classifying Braile characters [165] and recognizing spoken commands [66, 160, 166]. In these applications, metamaterials have the crucial advantage of requiring zero power. While a conventional intelligent device such as a Google Home^©^ or Alexa^©^ speaker consumes energy all the time (even when it is not being used), a speech-classifying metamaterial will detect commands by leveraging the acoustic wave physics, without consuming external power – satisfying a crucial requirement for edge computing applications. Designing elastic metamaterials with artificial intelligence is a prodigiously challenging task, due to the large and non-periodic nature of the required metamaterial geometries. Several approaches have emerged to solve this design problem. The first one is to use reservoir learning [161], where the metamaterial design is taken to be random, and the readout is optimised to produce the desired functionality. This approach has the advantage of producing convex optimisation problems. A second approach consists in simplifying the computational problem using a model reduction technique such as perturbative metamaterials, that reduces high-dimensional Finite Element problems to simpler mass-spring models with a few degrees of freedom [167, 168]. Finally, machine learning has also emerged as a tool to solve the hard design problems associated with intelligent metamaterials [169].

## 4 Acoustic metamaterials

One broad definition of acoustic metamaterials relies on the idea of using engineered internal structures, rather than constituent material properties, to control acoustic fields and waves in fluids. Early developments in this area focused on creating negative refractive index acoustic metamaterials [170, 171] and on finding anisotropic material properties needed for transformation acoustics and acoustic cloaking [172], [173], [174], [175]. A variety of different structural building blocks, including membrane-type metamaterials [176, 177], space-coiling structures [178, 179], and other advanced designs [180, 181], have since been developed for a wide range of applications that include acoustic beam shaping, impedance control, and sound absorption [9, 10, 12].

At present, manipulating sound with acoustic metamaterials remains a fast-growing subject with an increasing number of research directions, including the above-mentioned airborne acoustics at relatively low frequencies, the development of acoustic counterparts of phenomena found in other branches of physics, and novel avenues such as the control of ultrasound in water. These topics are overviewed in Section 4.1 with related applications described in Section 4.3. Subwavelength acoustic metastructures have recently formed a distinct class called acoustic metasurfaces and are thus discussed separately in Section 4.2.

### 4.1 From classic acoustics to novel phenomena

Current research on “stopping” sound by using acoustic metamaterials considers complex-structured designs. For instance, an octagonal onion-like structure with interconnected labyrinthine shells can reflect sub-kHz airborne sound in a cylindrical tube [182]. The 3D-printed samples have been shown to attenuate 67% of the incident sound on resonance with a volume filling fraction of only 13%, which makes them excellent candidates for sound mitigation applications. Other examples with similar and superior sound attenuation performance include space-coiled ventilated designs [183], tunable spider-web-inspired structures [184], porous metamaterials with reflected phase shifts [185], and metastructures with oblique-section nest resonators [186].

**Figure 3:**
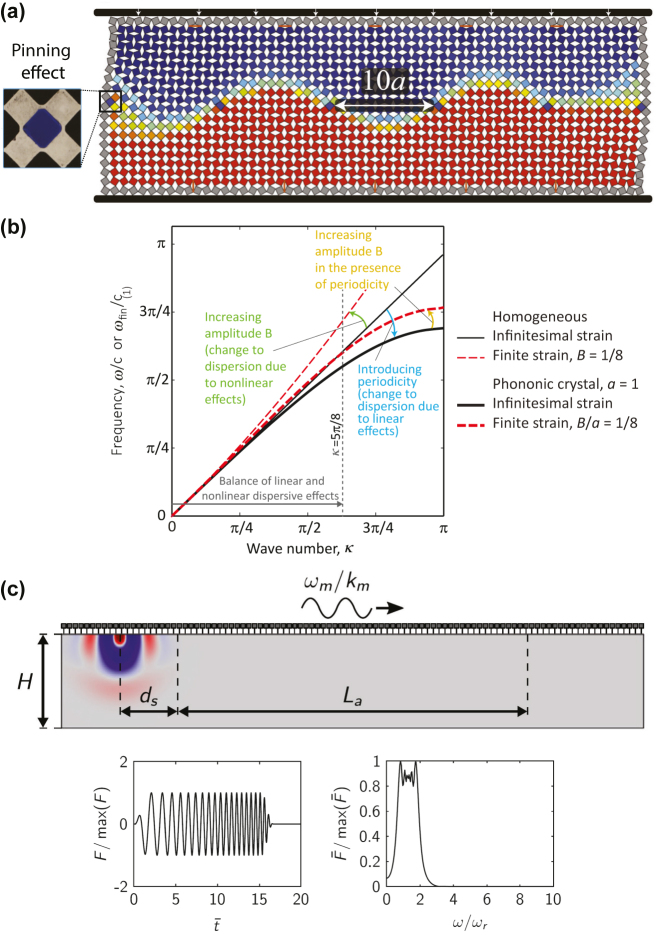
Nonlinear and time-varying effects in elastic metamaterials. (a) Numerically estimated deformation at vertically applied *ϵ* ^*yy*^ = −6% on a flexible metamaterial comprising 21 × 60 squares with localized pinning defects separated by 10 holes. (Adapted from [105]). (b) Wave dispersion in linear and nonlinear homogeneous materials and in linear and nonlinear elastic metamaterials. The intensity of the finite-strain nonlinearity increases with the wave amplitude. (Adapted from Ref. [112] with permission from the Royal Society). (c) A numerical model for right-going surface waves with a right-going modulating wave with the sub-figures showing the time history and frequency content of the point force applied at the source. (Reprinted from Journal of Mechanics and Physics of Solids, vol. 145, A. Palermo, P. Celli, B. Yousefzadeh, et al., “Surface wave non-reciprocity via time-modulated metamaterials”, p. 104181, Copyright © 2020, with permission from Elsevier).

**Figure 4:**
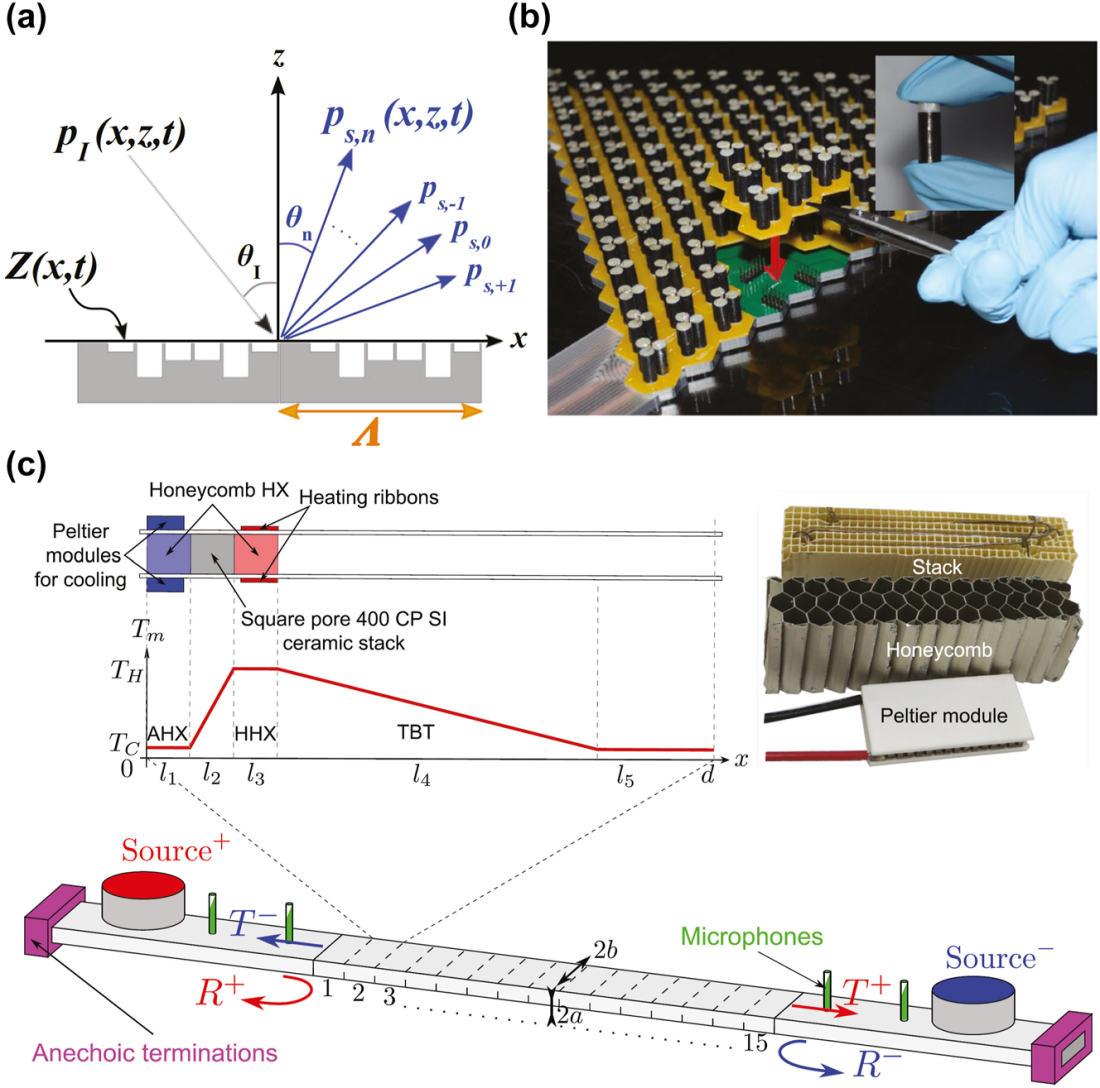
Acoustic metamaterials and metasurfaces. (a) Schematic representation of a diffuser with spatiotemporally modulated input impedance and the associated incident and scattered plane wave fields. (Published in Ref. [189]). (b) Fabricated topological WG insulator with a triangular domain wall. The inset shows the photograph of the cylinder trimers wrapped with a CNT film. (Material from Figure 3 in Hu, B., Zhang, Z., Zhang, H. et al. Non-Hermitian topological whispering gallery, Nature, published 2021, publisher – Nature, ISSN 1476–4687). (c) Thermoacoustic amplifier: (left-top) scaled representation of the unit cell with the temperature distribution indicated by colors; (right-top) the photography of the elements of the thermoacoustic cell and (bottom) a schematic view of the experimental setup for measuring the scattering matrix of the thermoacoustic amplifier. (Reprinted from Physical Review B, vol. 104, C. Olivier, G. Poignand, et al., “Nonreciprocal and even Willis couplings in periodic thermoacoustic amplifiers”, p. 184109, Copyright © 2021, Publisher – APS Physics).

Promising acoustic metamaterials are sound diffusers, which are structures designed to scatter incident plane acoustic waves equally in all directions [187]. Sound diffusers are conventionally used to minimize undesired aspects of the sound field, e.g., echoes in large spaces or standing waves in small enclosures. Traditional sound diffusers, such as the quadratic residue diffuser (QRD), are quasi-random phase gratings attached to reflecting surfaces, the purpose of which is to augment the spatiotemporal incoherence of the acoustic field scattered from reflective surfaces [187]. Early designs of a diffuser proposed by Schroeder [188] consisted of acoustic wells of varying depths, whose performance is limited in practice since scattered energy is unevenly distributed in space owing to minima between grating lobes associated with the periodicity of the acoustic wells. To address this drawback and improve sound diffusion, very recent work [189] proposes spatiotemporal modulation of a phase grating that is inspired by recent advances in spatiotemporal modulation of material properties used as a means to generate nonreciprocal wave propagation in electromagnetic, acoustic, and elastic domains [190], [191], [192]. The scattering amplitudes for all sound diffraction modes and the modulation harmonics can be obtained by satisfying the impedance relationship between the normal particle velocity and the local sound pressure, and the momentum equation at the diffuser surface. When the surface impedance varies as a function of time and space, the resulting scattered field includes harmonic frequency and wavenumber pairs associated with the spatiotemporal modulation (Figure 4a). As a result, the sound field scattered from the modulated diffuser increases temporal incoherence with the incident field and improves the spatial distribution of acoustic energy when compared with the same design without modulation. Semi-analytical and finite-element simulations show that the diffusion coefficient, a metric for the sound diffusivity, for the modulated quadratic residual diffuser is significantly improved in comparison to an unmodulated diffuser with the same geometry [189].

Intense research efforts have also been focused on exploring non-Hermitian fluctuations [193], [194], [195], [196], topological acoustics [197], [198], [199], and time-varying modulation [200], [201], [202], acoustic systems with fine-tuned gain and loss, facilitating unidirectional invisibility and exotic characteristics of exceptional points [121, 203, 204]. Likewise, the surge in other areas of physics in using topological insulators comprising non-trivial symmetry-protected phases has laid the groundwork in reshaping highly unconventional avenues for robust and reflection-free guiding and steering of both sound and light [205], [206], [207].

Along these lines, the research team led by Y. Cheng and J. Christensen has realized a topological gallery insulator using sonic crystals made of thermoplastic rods decorated with carbon nanotube films (Figure 4b), which act as a sonic gain medium by virtue of electro-thermo-acoustic coupling [208]. The word “gallery” refers to so-called whispering-gallery waves that were discovered by Lord Rayleigh in 1878 as sound waves creeping around the curved gallery of St Paul’s Cathedral in London. By engineering specific non-Hermiticity textures to the activated rods, it is possible to break the chiral symmetry of the whispering-gallery modes thus enabling the out-coupling of topological “audio-lasing” modes with the desired handedness [208].

Other flourishing topics in acoustic metamaterials are breaking sound wave reciprocity and Willis coupling. Reciprocity is broken when swapping source and receiver have non-identical frequency responses. It has been implemented by using spatiotemporal-dependent material properties [209], by combining nonlinear properties with an asymmetry [210], or introducing an external bias [211]. The external-bias approach, however, often neglects thermoacoustic effects that describe thermal interactions between acoustic waves and surrounding walls [212]. The Willis coupling parameters couple the potential and kinetic energies in the acoustic conservation relations, similarly to those for their electromagnetic counterparts, which were first described in the seminal work of Ref. [213]. This enables the enhancement of the ability to control waves in acoustic metamaterials compared to other materials that do not exhibit such coupling.

Nonreciprocal Willis coupling can be beneficial for developing thermoacoustic amplifiers. Specifically, for a 1D periodic thermoacoustic amplifier (Figure 4c), it is possible to derive closed-form expressions for the effective properties by using Padé’s approximation of the total transfer matrix [214]. The latter links the state vectors at both sides of the unit cell, and directly provides both even and non-reciprocal Willis coupling terms. In the first-order Taylor expansion of the transfer matrix elements, the odd Willis coupling is absent, while the even Willis coupling is found to be only related to the asymmetry of the temperature gradient that is applied to the unit cell. Even and nonreciprocal Willis couplings are found of equal modulus but an opposite sign and are almost purely imaginary at low frequencies, thus enabling a zero-group-velocity point and the opening of an amplification band at vanishing frequencies. Hence, the system effectively possesses a coalescence point in *k*-space that resembles a PT-broken phase. The effective parameters and scattering properties of single-unit and 15-unit systems have been validated experimentally, thus promoting thermoacoustics as an excellent means for designing non-reciprocal systems [215].

Similarly to the case of elastic metamaterials, one can achieve a roton-like dispersion in acoustic metamaterials by tailoring beyond-nearest-neighbor interactions [216]. Roton-like dispersion inside the first Brillouin zone can be obtained if the beyond-nearest-neighbor interactions dominate over the nearest-neighbor interactions. Then, for a certain range of positive wavenumbers, the partial wave mediated by the beyond-nearest-neighbor interactions becomes a dominant backward wave that leads to a net negative energy flow represented by a negative slope of the dispersion curve, which is related to the roton minimum.

Further developments of acoustic metamaterials are targeted to control high-frequency ultrasound in water, where one of the goals is developing new approaches for acoustic tweezers which can trap and manipulate small particles [217, 218]. Acoustic tweezers typically use multiple sources to create standing wave patterns in the water that can trap and move objects in ways constrained by the limited complexity of the acoustic wave field, which can be generated from an array of simple sources that are generally removed from the area of trapping and manipulation. A 3D acoustic tweezer proposed recently uses a single transducer and a PDMS acoustic lens [217], so that 3D trapping is achieved by combining the radiation force for trapping in two dimensions with the streaming force to provide levitation in the third dimension. The experimentally achieved levitation force reaches three orders of magnitude larger than for previous 3D trapping. Alternatively, spatially complex particle trapping and manipulation inside a boundary-free chamber can be obtained by using a single pair of sources and an engineered structure outside the chamber called a shadow waveguide [218]. The shadow waveguide creates a tightly confined, spatially complex acoustic field inside the chamber without requiring any interior structure that would interfere with net flow or transport. Particle trapping, particle manipulation and transport, and an acoustic analog of Thouless pumping have been experimentally demonstrated.

### 4.2 Acoustic metasurfaces

Acoustic metasurfaces, the airborne acoustics analogy of elastic metasurfaces, are subwavelength thickness structures allowing the control of sound waves in fluids [29, 219]. Their realization is a topic of continuous interest because of their compact sizes that enable manipulating large wavelengths of acoustic waves at audible frequencies that range from 20 Hz to 20 kHz.

Many acoustic metasurfaces contain Helmholtz resonators (HR), which are well-known devices for subwavelength manipulation of sound [220], [221], [222]. Yet, the phase shift effect introduced by each HR in an array is often considered independently of the others, thus neglecting possible coupling between neighboring HRs. Such a coupling can, however, be crucial for the ability to absorb and control the bandwidth at low sonic frequencies [223]. Therefore, recent studies have carefully analyzed the interaction between twin spherical HRs as a function of their separation and demonstrated a modal splitting into two resonances of different symmetry [224]. The underlying physical mechanism is characteristic of a degeneracy lifting resulting from the coupling between the two HRs, which leads to the occurrence of two modes: symmetric and antisymmetric with respect to the mirror plane between the two HRs. The distance between the HRs can be adjusted to tune the resonance frequencies of the modes, according to a specific aim. The theoretical calculations have been shown to be in good agreement with experimental measurements [224].

Other metasurface designs use surface wave phenomena to convert a near-field source to an energy focus in the far field, above a metasurface. This idea is inspired by recent analogous devices in electromagnetism [225, 226] and elasticity [227], which leverage the process known as Umklapp diffraction. The acoustic counterpart relies on a similar physical mechanism: a designed metasurface comprises two regions where guided or trapped surface modes exist, and at the interface, between the regions, a scattering process occurs whereby energy is diffracted out of the metasurface states into the far field. The mode dispersion in each region of the surface is carefully designed; (1) to ensure the scattering process allows the Umklapp-like diffraction of the surface mode, and (2) to maximize the mode-shape overlap and minimize the impedance mismatch between regions. The analyzed systems are radially symmetric so that the overlap of diffracted beams in the far field results in an enhanced focal point.

In particular, the acoustic metasurface in this application can be represented by an aluminium plate with a periodically modulated thickness profile submerged in water [228]. The periodic structure is designed to modify the dispersion of the coupled Scholte modes that would be present on an otherwise unpatterned plate in water. When excited with a point-like (monopole) acoustic source at the plate centre, Scholte modes are excited in the first region and undergo diffraction at the transition to the second region, radiating into the fluid. Finite-element simulation and experiments reveal the existence of frequency-dependent focal spots on both sides of the sample, no matter which side the source is placed on. It is therefore possible to focus sound on the flat side of the metasurface due to the coupling and modulation of the Scholte modes.

### 4.3 Applications of acoustic metamaterials

Potential applications of acoustic metamaterials and metasurfaces have a broad spectrum ranging from sound attenuation [9, 10, 219, 229, 230] to exotic acoustic camouflage [172, 174] and analog computing [12]. Employing random medium theory on acoustic metasurfaces is another promising route for controlling sound fields in a room environment (i.e., diffuse sound fields) [189, 231]. Among the designs described above, metastructures with large Willis coupling pave the way for a better physical understanding of Willis couplings in non-reciprocal systems, to facilitate engineering applications of Willis materials, and for various applications of such systems to further control acoustic waves at very low frequencies [214, 215]. Recently, topological studies of acoustic metasurfaces have been carried out in depth. The three key research branches include non-Hermitian, nonlinear, and non-Abelian braiding, each of which delivers many interesting results. For instance, non-Abelian braiding of two or three modes enables the implementation of quantum logic operations [232]. Acoustic tweezers allow contact-free, bio-compatible, and precise manipulation of particles from millimeter to sub-micrometer scales [217, 218], while acoustic metasurfaces can be used in underwater sensing or nondestructive testing and evaluation [228].

Other emerging and very promising application directions are acoustic imaging and medical therapy, e.g., therapeutic applications of focused ultrasound [233]. Thanks to the ability of acoustic metamaterials to accurately engineer wavefronts, one can generate acoustic holograms by synthesizing acoustic images that shape the areas where mechanical waves have high amplitudes and the areas where the medium is at rest. Compared to light, acoustic waves can penetrate deeper into biological tissues, thus enabling the shaping and focusing of ultrasonic fields inside biological media. For instance, accurately designed metastructures can encode complex wavefronts to compensate for the phase aberrations produced by stiff layers of tissues, such as skull bones in transcranial propagation, and allow the creation of arbitrary therapeutic patterns in the brain or even vortex beams.

Recent works have demonstrated the potential of acoustic holograms to deliver therapeutical acoustic images for the non-invasive treatment of neurological disorders [233, 234], and to produce cavitation [235, 236] and thermal [237] patterns. When targeting acoustic beams into the brain, accurate focusing is mainly limited due to the strong phase aberrations produced by the refraction and attenuation of the skull. Acoustic holographic lenses overcome these limitations by synthesizing aberration-free ultrasonic fields of complex spatial distribution inside the skull. By using low-cost 3D-printed lenses, ultrasonic beams can be focused not only on a single point but be made to overlap at one or various target structures simultaneously, e.g., left and right hippocampi. Other complex fields such as vortex beams for trapping small objects can also be obtained. The technique was tested to perform localized drug delivery in the brain of a small laboratory animal. In addition, acoustic holograms encode the multiple reflections inside the skull, increasing the angular spectrum of the acoustic image. The technique was also tested to shape thermal patterns in soft tissue to raise the temperature locally [237].

These results open new paths for emerging therapeutic ultrasound applications in neurology, including blood-brain barrier opening for localized drug delivery or neuromodulation. On the other hand, increasing the temperature using low-cost and MRI-compatible holographic transducers might be of great interest for ultrasound hyperthermia, physiotherapy, or high-intensity focused ultrasound, where the control of specific thermal patterns is needed.

## 5 Mechanical metamaterials

Mechanical metamaterials form yet another subcategory of architected materials, in this case materials with unprecedented mechanical properties or functionalities. Similar to elastic and acoustic metamaterials, these properties originate from an interplay between the rational design of their 2D or 3D (micro)architectures and their constituent materials [13]. Examples include auxetic (also known as negative Poisson’s ratio) [238], shape-matching [239], action-at-a-distance [240], programmable [241, 242], adaptable [243], tunable elastic properties [244] and crumpled-based metamaterials [245].

### 5.1 Design approaches and functionalities

The architecture of mechanical metamaterials can be represented by either regular repeating unit cells or random microstructures, each of which have their own pros and cons, and govern unconventional behavior on the macroscale. One of the main challenges in the design of mechanical metamaterials is to find a proper combination of microstructural features that can ensure structural integrity and, simultaneously, deliver desired mechanical behavior. Other challenges include achieving tunable performance, e.g., by varying external loads or environmental conditions, and multi-functionality (e.g., double auxeticity, load- or temperature-dependent response, etc.) that allows combining several functionalities by using a single microstructure.

**Figure 5:**
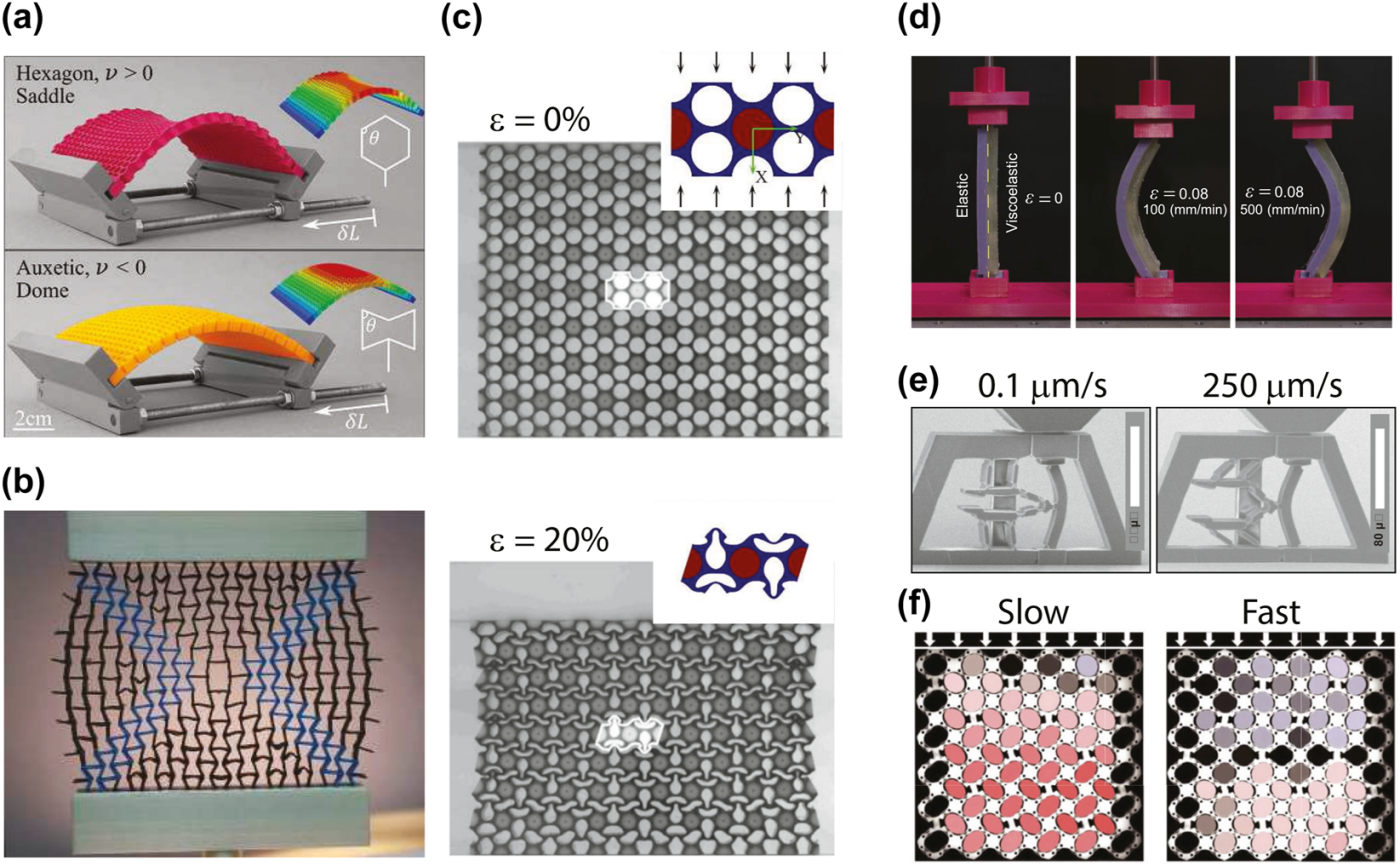
Mechanical metamaterials. (a) By controlling the values of Poisson’s ratio, one can control the out-of-plane curvature of meta-plates (adapted from M. J. Mirzaali, et al., Advanced Materials, 33(30), p. 2008082 (2021)); (b) a wider range of properties can be achieved by intruding multiple materials in the structure of the mechanical metamaterials (Reprinted from M. J. Mirzaali et al. “Multi-material 3D printed mechanical metamaterials: Rational design of elastic properties through spatial distribution of hard and soft phases”, Appl. Phys. Lett. 113, 241903 (2018) with the permission of AIP Publishing); (c) numerical and experimental images of a multiphase composite with stiff inclusions and voids in an undeformed state and loaded in the *x* direction (adapted from J. Li, V. Slesarenko and S. Rudykh, Soft Matter, 14, 6171 (2018); (d) viscoelastic bi-beams buckle to left and right in response to the applied strain rate (adapted from S. Janbaz, et al., Science Advances, 6(25), p. eaba0616 (2020)); (e) A viscoelastic micro gripper powered by a bi-beam (adapted from S. Janbaz, et al., arXiv:2206.15168); (f) viscoelastic oligomodal metamaterials exhibit different global buckling modes once compressed slow or fast (adapted from A. Bossart, et al., PNAS, 118(21), p. e2018610118 (2021)).

Flexible mechanical metamaterials, previously discussed in the context of nonlinear wave propagation (Section 3.1), can be designed to show shape-matching and auxetic behavior in the (quasi-) static regime. These are examples of unusual properties that can be obtained by tailoring mechanical characteristics, e.g., elastic stiffness and Poisson’s ratio, through modifying geometric features at the unit-cell level. For instance, cellular structures formed by a purposely designed combination of auxetic and conventional unit cells can match a predefined shape upon deformation [239, 246, 247] or exhibit intrinsic curvature (Figure 5a) [248]. Note that despite different geometries, the spatial arrangement of the unit cells remains regular.

Alternatively, one can harness randomness as a design tool to expand the design space of mechanical metamaterials for achieving even more exotic properties. It has recently been shown that disordered architectures of lattice-unrestricted networks exhibit a high probability of auxetic and double-auxetic behavior, also in the stretch-dominated domain [249, 250]. The latter feature uncovers the potential of these networks in developing stiff metamaterials with extremely negative (or positive) Poisson’s ratios [249]. Patterned randomness can also be used to independently tailor the elastic modulus and Poisson’s ratio of a mechanical metamaterial [251] in addition to the known tuning-by-pruning approach [252].

Other tools to expand the design space of mechanical metamaterials include the use of multiple constituent materials, harnessing elastic instabilities, and the exploitation of nonelastic material behavior. These approaches enable properties that are impossible or challenging for single-material configurations and break the limits imposed by elastic regimes. Below, we overview these tools in more detail.

Multi-material mechanical metamaterials benefit from rapid advances in multi-material additive manufacturing that have enabled the production of truly intricate designs at various size scales. Recent works have shown that combining hard and soft materials in 2D or 3D arrangements can be used to circumvent intrinsic limitations (i.e., in terms of elastic properties) of metamaterials made of a single material. A remarkable example is a random assignment of a hard phase to soft cellular structures that allows the extension of the ranges in the elastic modulus-Poisson’s ratio plane [253]. Toward this aim, artificial neural networks have been considered as a powerful tool that may help designers bypass “trial-and-error” approaches and speed up the process of extracting corresponding (micro-)architectures and material distributions for any desired target properties [254]. On the other hand, patterned designs of the hard phase can be effective for independent tuning of the elastic properties (Figure 5b) [253]. The deposition of the second hard phase in the microstructure of mechanical metamaterials can also create local non-affine deformations which can be correlated to the values of the Poisson’s ratio [255].

Switchable properties in microstructured metamaterials can be achieved by harnessing elastic instabilities – a phenomenon that plays an important role in pattern formations in soft biological systems [256]. Such systems typically combine a soft matrix and a stiff phase (such as fibers or inclusions). In the 2D setting, stiffer layers undergo buckling when the composite is compressed beyond the critical level [257]. The larger the layer stiffness contrast and concentration of the stiffer phase, the larger the critical wavelength that allows predesigning switchable metamaterial microstructures. Furthermore, 3D-printed soft layered composites with a non-dilute stiff phase can form a twinning microstructure with finite-size anti-symmetric domains by developing long-wave instability upon exceeding the critical value of the compressive strain along the layers. The obtained finite-size regulated domain pattern distinctly differs from a classical wrinkling [258]. The instability phenomenon can be used to induce a negative group velocity state in non-periodic composites, potentially enabling left-handed behavior and negative energy transfer.

Three-dimensional fiber composites, similarly, undergo buckling when compressed, but are more stable and require higher strains to trigger buckling [259]. Interestingly, the buckling plane orientation can be controlled by the in-plane periodicity of fiber arrangements, together with material parameters [260]. Composites with periodic stiff inclusions in a soft elastomer exhibit domain formations and pattern transitions under large deformations [261]. Instabilities in multiphase composites with stiff inclusions and voids periodically distributed in a soft matrix can induce the rearrangement of new microstructural morphologies (Figure 5c) [262]. Such pattern transformations are accompanied by void collapse that results in auxetic behavior, while the stiff inclusions regulate the onset of instabilities and the post-buckling transformations. Hence, the auxetic behavior can be pre-designed and achieved earlier, at smaller strains, and distinct post-buckling patterns can be induced through the positioning of stiff inclusions [263].

Other material properties – such as viscoelasticity – can be used to increase the pool of admissible microstructures upon instability. For instance, identical samples can develop different patterns due to different loading histories, as shown experimentally on 3D-printed laminates loaded at different strain rates [264]. Viscoelasticity can also be useful to control the deformation modes in soft metamaterials that work on the basis of mechanical instabilities [265, 266]. This can be achieved in two ways, both of which imply that viscoelasticity effectively seeds imperfections required for switching the response of a metamaterial depending on an applied strain rate. The first approach relies on the reduction of the navigation of instability patterns by designing a simple building block, such as a bi-beam [267]. The bi-beams are formed by lateral attachment of two flexible beams made from two polymers with highly different viscoelastic properties (Figure 5d) so that one of the beams is almost strain-rate insensitive, and the other is highly viscoelastic. This enables a reliable strain rate-dependent control of buckling direction as a principle to design multifunctional metamaterials. For example, this idea is applied to construct flexible structures with switchable auxetic-non-auxetic behavior or to show apparent negative viscoelasticity (i.e., where the apparent stiffness is lower for larger loading rates and larger for lower loading rates) [267]. Taking into account that the commercial 3D printing of such strain-rate-dependent materials might be limited due to the lack of 3D printable viscoelastic polymers, one can use purposeful geometrical imperfections to induce a robust and highly predictable strain-rate-dependent behavior. For example, the curved geometry of a bi-beam (that actuates a compliant micro-gripper, Figure 5d) can overcome the limits of single-material printing using two-photon polymerization [268]. In this case, once differential curing of a photo-polymer provides different levels of viscoelasticity at the two sides of a beam, the low-power cured layer remains always softer than the one cured with a higher power. The curved geometry of the beam can serve as an imperfection that combined with material-based imperfection form strain-rate dependency.

The second approach uses viscoelasticity to control minimum energy pathways in combinatorial designs such as oligomodal metamaterials [265, 269]. Oligomodal metamaterials are metamaterials that feature a constant number (>1) of soft deformation modes, regardless of system size [269]. Each of these modes spans the entire design domain [269]. With a rational selection of elastic and viscoelastic joints within their structures they exhibit strain-rate dependent mode selection (Figure 5e) [269]. While comparing with kinematic systems with many soft modes, viscoelastic oligomodal designs transform their properties (e.g., Poisson’s ratio) simply by changing the applied strain rate using a simple loading scheme. Alternatively, one can also use viscoelasticity to switch between buckling or frustration (Figure 5f) [266]. A similar method, using intermediate strain rates, can also be used to generate extreme shock absorption by delaying both buckling and unbuckling [266]. In general, viscoelasticity elaborates the design space of metamaterials to exhibit switchable responses that pave the way toward advanced materials and devices.

### 5.2 Applications of mechanical metamaterials

The majority of the described mechanical metamaterials have complex architectures that can be realized almost exclusively by means of additive manufacturing technologies, e.g., fused deposition modeling [239, 240, 248], two-photon polymerization [268], stereolithography [13, 247], material jetting [242, 248, 264, 267], etc. Often, a special material or a combination of such materials is required to enable the designed mechanical properties or functionalities [239, 262, 267, 269]. Therefore, further advances in the development of additive manufacturing techniques, including better resolution and inter-layer adhesion, lower porosity, broader ranges of base materials, etc., will promote wider applications of mechanical metamaterials.

Mechanical metamaterials have already shown promising potential for numerous applications, including the design and fabrication of programmable matter, soft robotics, biomedical devices, such as implants or exoskeletal prostheses, wearable electronics, smart clothing, shoes, etc. For instance, a proper combination of conventional and auxetic unit cells can be used to design an orthopedic meta-implant that in contrast to a conventional counterpart does not retract from a bone under biomechanical loading [270]. The rational design of programmable metamaterials [242, 271] enables control of the actuation pattern and actuation characteristics in the fabrication of the metamaterials, thereby eliminating the need for networks of distributed actuators, sensors, and controllers that are usually needed to achieve complex actuation. Adaptable metamaterials with double curvatures can be used to fit a shape curvature required for soft wearable devices [248]. Meta-plates with cellular [248] or kirigami-based [272] architectures are promising for the design of adaptive mirrors capable of controlling focused laser beams of incoming wave fronts. Finally, by further extending control over buckling modes by means of viscoelasticity, one may promote the design of mechanical metamaterials in the field of complex strain rate-dependent machines. Hence, it can be anticipated that the fabrication of even more complex 3D metamaterials and miniaturized machine-like devices is not far from realization.

## 6 Conclusions and outlook

We have seen throughout this broad review that the general domains of nanophononics and elastic, acoustic, and mechanical metamaterials, despite their now relatively mature history, still contain many unsolved problems and forthcoming challenges whose solution deserves the attention and collaborative efforts of the scientific community across multiple disciplines.

The broad scalability of the fundamental equations of mechanics and acoustics, which is relatively rare in other branches of physics, shows that similar phenomena can be applied across a broad spectrum of problems ranging from control of light–matter interactions with phonons of nanometric wavelengths to control of seismic waves of several hundred meters in wavelength. For this reason, even in a small workshop like the EUROMECH Colloquium summarized in this review, one can observe the value of interaction between scientists from different areas. For example, in this workshop nanochemists working on the fabrication of optomechanical cavities were sharing common interests with civil engineers working on sound isolation for buildings.

In this review, we have also shown that new theoretical concepts are still emerging and that more powerful computational tools are in demand to help elucidate the physics of the complex material systems that are continuously being proposed or discovered. Equally important, advanced experimental demonstrations continue to emerge, often inspired by the new observed phenomena. This is all driven by the growing number of applications, especially after the recent rise of tunable and reconfigurable metastructures and other novel concepts. As extraordinary as the current applications are, many more are awaiting in the future.
